# Lupeol, a Plant-Derived Triterpenoid, Protects Mice Brains against Aβ-Induced Oxidative Stress and Neurodegeneration

**DOI:** 10.3390/biomedicines8100380

**Published:** 2020-09-26

**Authors:** Riaz Ahmad, Amjad Khan, Hyeon Jin Lee, Inayat Ur Rehman, Ibrahim Khan, Sayed Ibrar Alam, Myeong Ok Kim

**Affiliations:** Division of Life Sciences and Applied Life Science (BK 21plus), College of Natural Science, Gyeongsang National University, Jinju 52828, Korea; riazk0499@gnu.ac.kr (R.A.); amjadkhan@gnu.ac.kr (A.K.); dlguswls363@naver.com (H.J.L.); inayaturrehman201516@gnu.ac.kr (I.U.R.); ibrahimbiotech11@gmail.com (I.K.); ibrar@gnu.ac.kr (S.I.A.)

**Keywords:** Alzheimer’s disease, reactive oxygen species (ROS), neuroinflammation, neurodegeneration, cognitive dysfunction

## Abstract

Alzheimer’s disease (AD) is a progressive neurodegenerative disorder that represents 60–70% of all dementia cases. AD is characterized by the formation and accumulation of amyloid-beta (Aβ) plaques, neurofibrillary tangles, and neuronal cell loss. Further accumulation of Aβ in the brain induces oxidative stress, neuroinflammation, and synaptic and memory dysfunction. In this study, we investigated the antioxidant and neuroprotective effects of the natural triterpenoid lupeol in the Aβ_1–42_ mouse model of AD. An Intracerebroventricular injection (i.c.v.) of Aβ (3 µL/5 min/mouse) into the brain of a mouse increased the reactive oxygen species (ROS) levels, neuroinflammation, and memory and cognitive dysfunction. The oral administration of lupeol at a dose of 50 mg/kg for two weeks significantly decreased the oxidative stress, neuroinflammation, and memory impairments. Lupeol decreased the oxidative stress via the activation of nuclear factor erythroid 2-related factor-2 (Nrf-2) and heme oxygenase-1 (HO-1) in the brain of adult mice. Moreover, lupeol treatment prevented neuroinflammation by suppressing activated glial cells and inflammatory mediators. Additionally, lupeol treatment significantly decreased the accumulation of Aβ and beta-secretase-1 (BACE-1) expression and enhanced the memory and cognitive function in the Aβ-mouse model of AD. To the best of our knowledge, this is the first study to investigate the anti-oxidative and neuroprotective effects of lupeol against Aβ_1–42_-induced neurotoxicity. Our findings suggest that lupeol could serve as a novel, promising, and accessible neuroprotective agent against progressive neurodegenerative diseases such as AD.

## 1. Introduction

Neurodegenerative diseases are incurable conditions that result in the progressive loss of neuronal cells. There are several neurodegenerative disorders including Alzheimer’s disease (AD), which is the most common and represents approximately 60–70% of all dementia cases [1,2]. AD is a chronic and progressive neurodegenerative disorder that affects synaptic and cognitive functions. The pathophysiology of AD is the formation and accumulation of extracellular amyloid-beta (Aβ), plaques, intracellular neurofibrillary tangles, and a loss of connection among the nerve cells in the brain [3,4,5] Aβ-peptide is generated from a transmembrane protein called the amyloid precursor protein (APP) by the action of a beta secretase-1 enzyme (BACE-1) [6,7]. The increased activity of these enzymes is responsible for the sequential cleavage of APP, resulting in the formation and aggregation of the Aβ-peptide [7,8].

The accumulation of Aβ in the brain enhances oxidative stress and neuroinflammation and affects the memory function of the brain [9]. The elevated level of reactive oxygen species (ROS) disrupts the normal functioning of various biomolecules (lipid and DNA) in the brain [10,11]. The higher oxidative stress is responsible for the progression of the pathophysiology of AD by several mechanisms such as the activation of the innate immune system and the release of inflammatory mediators. Boosting the antioxidant defense mechanisms may counteract the progression of AD and its consequences.

Aβ and oxidative stress in the brain are responsible for the activation of glial cells, which are involved in the production of inflammatory cytokines and mediators, resulting in neuroinflammation [12]. The activated glial cells are an important component of chronic neuroinflammation, neuronal loss, and the progression of AD [13,14]. Increased levels of oxidative stress and neuroinflammation disturb the proper structure and function of neurons as well as the synaptic, memory, and cognitive function of the brain [15,16].

Natural products played important role in human disease therapy. Terpenoids, also known as terpenes are the largest group belonging from natural compounds synthesized in plants [17,18]. Among them, triterpenoids are a highly diverse group of natural products broadly distributed in plants [19]. The majority of the known triterpenoids arise from the dammarenyl cation [20] having broad range of biological activities including anti-inflammatory, anti-tumour, anti-HIV antiviral, insecticidal activities and for the treatment of metabolic diseases [21].

Lupeol is a pentacyclic triterpenoid and biologically active compound, naturally found in fruits, vegetables, and several medicinal plants [22]. In vegetables, it is mainly found in white cabbage, pepper, cucumber, and tomato, and in fruits, it is mainly found in mango, fig, strawberry, and red grapes [23]. Lupeol has a wide range of biological effects including anti-cancer, anti-microbial, anti-diabetic, cardio, and hepatoprotective effects [24]. Lupeol has also been shown to exhibit anti-oxidant and anti-inflammatory effects [25,26]. The purpose of the current study was to evaluate the effects of lupeol against Aβ_1–42_-induced oxidative stress-mediated neuroinflammation, neurodegeneration, and cognitive dysfunctions in mice. The amyloid-beta-induced AD mouse model is a known and accepted model of Alzheimer’s disease, produced by an intracerebroventricular injection of the amyloid-beta-peptide into the brain of mice [27]. The amyloid fibrils are formed from the Aβ peptide, which occurs in different forms of varying sizes. The Aβ_1–42_ represents the most expressed form in several types of AD cases. The Aβ_1–42_ accumulates to form mini dimers, oligomers, and other insoluble fibrils. To show all amyloid-beta forms, we injected the Aβ_1–42_ peptides into the brains of mice. For biochemical studies, we conducted western blot and immunofluorescence analysis for the Aβ_1–42_-mediated oxidative stress and neuroinflammation. For the behavioral analysis, we performed the Morris water maze (MWM) test and Y-maze tests.

## 2. Materials and Methods

### 2.1. Chemical

Aβ and lupeol (CAS Number*: 545-47-1) were purchased from Sigma Co. (St. Louis, MO, USA).

### 2.2. Animals

Wild-type male C57BL/12N mice (12 mice for western blot and immunofluorescence each) (n = 36) that were eight weeks old and 25–30 g in weight were purchased from Samtako Bio Usan South Korea. All mice were processed according to the protocol approved by the Animal Ethics Committee of the Division of Applied Life Sciences, Gyeongsang National University, South Korea (Approval ID: 125, 3 Jun 2020). Mice were adapted for one week in the university animal house to a 12 h light/dark cycle at 23–25 °C with 60 ± 10% humidity and were provided with standard food and water. We used male mice in this study, according to the literature, male mice are more resistant to stress, hard environment, and hormonal changes.

### 2.3. Drug Treatment

**Figure 1:**
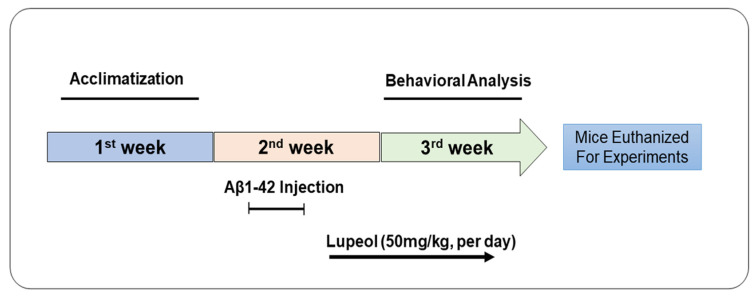
Schematic diagram of experimental design showing duration of lupeol treatment to Aβ mouse model of Alzheimer’s disease and their behavioral analysis.

The mice were randomly divided into three groups, and the mice received the treatment as described in Figure 1. The Aβ_1–42_ peptide of human origin was reconstituted in sterile saline solution as a stock solution at a concentration of 1 mg/mL, followed by incubation at 37 °C for four days. Stereotaxically Aβ_1–42_ peptide aggregates or a vehicle (0.9%NaCl, 3 µL/5 min/mouse) were injected into the ventricles (i.c.v.), by using a Hamilton micro-syringe, 2.4 mm dorsoventral (DV), 0.2 mm anteroposterior (AP), and 1 mm mediolateral (ML) to the bregma. After 24 h of i.c.v. Aβ_1–42_ and the vehicle, mice were divided into the following groups: (1) The control group, which received saline for two weeks as a vehicle; (2) the Aβ_1–42_ group; and (3) and the Aβ_1–42_ + lupeol (50 mg/kg/day/mice/p.o.) group. Dosages of lupeol were selected following previously published studies [28]. The lupeol alone group was not considered in the current study, as previously no unwanted effects of lupeol have been reported in the brain [29]. Lupeol was dissolved in dimethyl sulfoxide (DMSO) to prepare a stock solution. Each day, fresh lupeol solution was prepared in normal saline, according to the required volume of injection, and was employed to treat the mice.

### 2.4. Behavior Studies

To analyze the effect of lupeol on memory function, we performed the Morris water maze (MWM) test and the Y-maze test. The MWM equipment consisted of a round tank (100 cm in diameter, 40 cm high, and 15.5 cm deep) filled with water, which was made opaque with white ink. A transparent platform with a diameter of 10 cm and a height of 20 cm was hidden 1 cm below the surface of the water in one quadrant of the apparatus during the experiment. The MWM test was carried out for four days, with each mouse being trained by using a hidden platform in one quadrant with three quadrants starting to rotate. After the training session, a probe test was conducted by removing the hidden platform and the mice were allowed to swim freely for 60 s in the water tank. In the probe trial, the number of crossings over the hidden platform and the time spent in the area where the hidden platform was present were calculated. All of the data were recorded using video-tracking software (SMART, Panlab Harvard Apparatus Bioscience Company, MA, USA).

For the evaluation of spatial working memory, we performed the Y-maze test, which was built of black painted plastic. Each arm of the maze was 50 cm long, 20 cm high, and 10 cm wide at both the bottom and top. The mouse was placed at the center of the apparatus and allowed to explore the apparatus for 8 min. The number of arm entries was observed visually. A spontaneous alteration was defined as the successive entry of the mice in three arms in an overlapping set of triplets. The percentage (%) of spontaneous alternation behavior was calculated as [successive triplet sets (entries in three different arms consecutively)/total number of arm entries − 2] × 100. A higher percentage of spontaneous alternation behavior was considered for showing the improved spatial working memory and vice versa.

### 2.5. Protein Extraction and Homogenization of the Brain of Mice

After the behavioral study, all mice were anesthetized with ketamine/xylazine and the brain tissues were immediately removed and the cortex and hippocampus were separated. The tissues were homogenized in a PRO-PREP^TM^ extraction solution (iNtRON Biotechnology, Dallas Texas MA USA) and centrifuged at a speed of 13,000 rpm for 25 min at 4 °C. The supernatant was collected and stored at −80 °C.

### 2.6. Western Blot Analysis

Western blotting was performed as described previously, with some modification [30,31]. The protein concentrations were quantified by using a Bio-Rad Protein Assay Kit (Bio-Rad Laboratories, CA, USA). Equal amounts of protein samples (15–30 mg) were electrophoresed on a 12–15% SDS PAGE gel and transferred to a polyvinylidene difluoride (PVDF) membrane. A protein marker (GangNam-STAIN, iNtRON Biotechnology, CA USA) was loaded in parallel for the determination of the molecular weights of the proteins. To reduce the nonspecific bindings, the membranes were blocked in skim milk (5% *w*/*v* skim milk in 1X Tris-Buffered Saline, 0.1% Tween® 20 Detergent (1xTBST), and the membranes were then incubated with the required primary antibodies at 4 °C (1:1000 dilutions, as optimized) for 16 h. After the primary antibody treatment, the membranes were washed with 1× TBST and blocked with horseradish peroxidase-conjugated secondary antibodies, as appropriate. After washing, the bands were detected using an Enhanced chemiluminescent (ECL) detection reagent (EzWestLumiOne, ATTO, Tokyo, Japan). The optical densities of the bands were evaluated with ImageJ (v. 1.50, NIH, Bethesda, MD, USA).

### 2.7. Immunofluorescence

Immunofluorescence staining was performed as described previously [32,33]. After washing with 1% 1x Phosphate-Buffered Saline (PBS), the slides were treated with proteinase K for 5 min and incubated with a blocking solution (2% normal serum, 0.3% Triton X-100). After blocking, the slides were incubated with primary antibodies (1:100) for 24 h. After incubation with primary antibodies, slides were treated with fluorescein isothiocyanate (FITC) labeled secondary antibodies for 2 h. After the completion of the secondary antibody treatment, the slides were treated with 4,6-diamidino-2-phenylindole (DAPI), for visualizing the nucleus. The slides were then rinsed and mounted with coverslips by using a DAKO fluorescent mounting medium. The images were captured using FluoView 1000 (FV 1000 MPE). Through ImageJ, the relative integrated densities were evaluated among the different experimental groups, which sums all of the pixels within a region and gives a total value and the obtained values were compared among the different experimental groups.

### 2.8. Antibodies

The antibodies used in this study are given in Table 1.

### 2.9. Reactive Oxygen Species (ROS) ssay

The assay was performed to analyze the levels of Reactive Oxygen Species (ROS) in the brains of the experimental groups (n = 6 mice/group). The ROS assay was based on the oxidation of DCFH-DA) to 2′7′-dichlorofluorescein (DCF) [27]. The conversion of 2’-7’dichlorofluorescin diacetate (DCFH-DA to DCF was assessed by a spectrofluorometer at an excitation wavelength of 484 nm and an emission wavelength of 530 nm. To measure the conversion of DCFH-DA to DCF in the absence of homogenate (background fluorescence), parallel blanks were used. The ROS levels were quantified from a DCF standard curve and expressed as relative pmol DCF/mg protein.

### 2.10. Statistical Analysis

For the analysis of the intensities of the bands, the X-ray films were scanned, and through the ImageJ software (v. 1.50, NIH, Bethesda, MD, USA), the densities were measured. Similarly, for the immunofluorescence analysis, the integrated density was analyzed through ImageJ. The data were been presented as the mean ± standard error of the mean (SEM). For statistical analysis, one-way analysis of variance (ANOVA) followed by the Student’s “t” test was used for comparisons of the different groups. The graphs were generated via GraphPad Prism6 (GraphPad Software, San Diego, CA, USA). P values of less than 0.05 were considered to indicate a significant difference between the groups; * indicates a significant difference from the vehicle-treated control group, while # indicates a significant difference from the Aβ_1–42_ treated groups.

## 3. Results

### 3.1. Administration of Lupeol Reduced the Aβ and BACE-1 Expression

**Figure 2:**
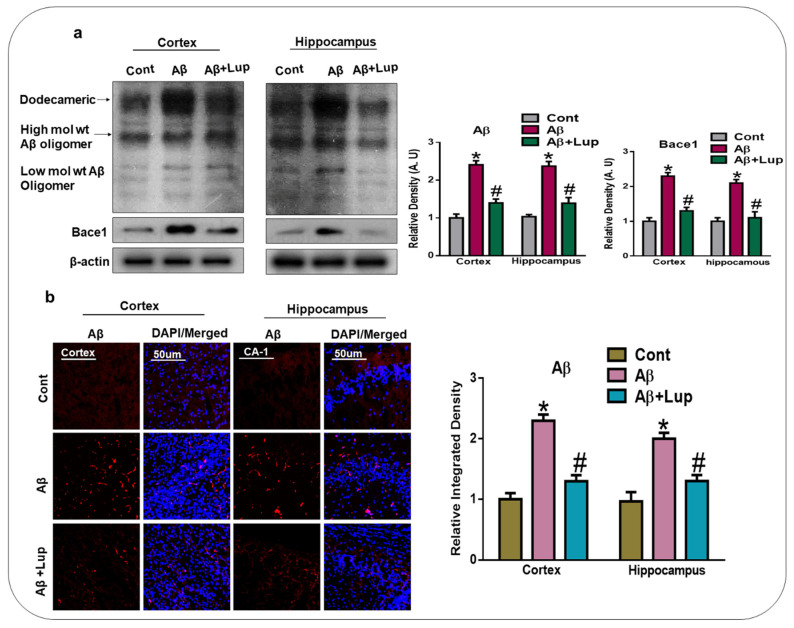
Administration of lupeol reduced the Aβ and BACE-1expression. (**a**) Western blot analysis and representative histogram of Aβ, and BACE-1 in the cortex and hippocampus of experimental mice. (n = 6 mice/group) the bands were quantified using ImageJ software, and the differences were represented by histograms. β-actin was used as a loading control. (**b**) Confocal microscopy of Aβ (n = 6/mice/group), red, with their representative histogram and stained with DAPI, blue in cortex and hippocampus (CA1 region), in the experimental mice, and are presented relative to the control. Magnification 10×. Scale bar = 50 µm. The expressed data are relative to the control. * significantly different from saline-injected; # significantly different from Aβ-injected. Significance = * *p* 0.05, # *p* 0.05. Aβ: Amyloid beta, BACE-1: beta-site amyloid precursor protein cleaving enzyme-1, Lup: Lupeol, DAPI: 4’, 6-Diamidino-2-Phenylindole, Dihydrochloride.

To analyze the effects of lupeol against the elevated amyloidogenic process, we analyzed the expression of Amyloid beta (Aβ) and beta amyloid cleaving enzyme-1) BACE-1 in the experimental groups. According to our findings, a single intracerebroventricular injection of Aβ_1–42_ increased the expression of Aβ and BACE-1 in the cortex and hippocampus of the mice brains, compared to the saline-treated control group, as shown by the western blot results. Treatment with lupeol significantly reduced the expression of Aβ and BACE-1 compared to Aβ_1–42_ injected mice (Figure 2a). We also analyzed the expression of Aβ through immunofluorescence, and the findings showed an increased immunoreactivity of Aβ in the cortex and hippocampus of Aβ_1–42_-treated mice compared to the control mice. The expression of Aβ was markedly reduced with the administration of lupeol compared to the Aβ_1–42_-injected mice (Figure 2b).

### 3.2. Oral Administration of Lupeol Decreased Oxidative Stress via the Nrf2/HO1 Signaling Pathway

**Figure 3:**
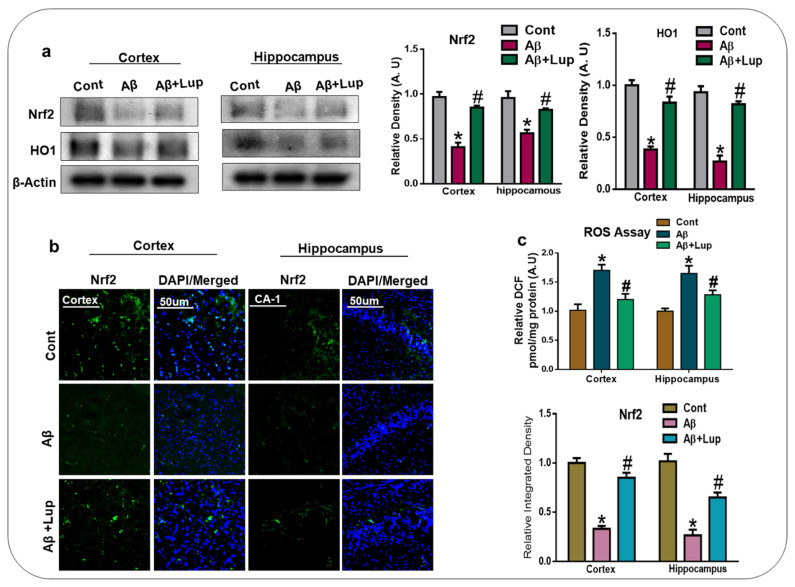
Oral administration of lupeol decreased oxidative stress via Nrf2/HO1 signaling pathway. (**a**) Western blot analysis of Nrf2/HO1, in the cortex and hippocampus of experimental mice (n = 6 mice/group). Western blot bands were quantified by ImageJ software, and the differences were represented by a histogram. β-actin was used as a loading control. (**b**) Immunofluorescence analysis of Nrf2 (green) along with their respective histogram stained with DAPI (blue) in cortex and hippocampus (CA1), in the adult mice (n = 6 mice/group). The data are presented relative to control. Magnification 10×. Scale bar = 50 µm. (**c**) is a representative histogram of the ROS level in the homogenates of the cortex and hippocampus of the adult mice. Aβ_1–42_ increased the levels of ROS, while treatment with lupeol decreased the level of ROS in the adult mice brain. The expressed data are relative to the control. * significantly different from saline-injected; # significantly different from Aβ-injected. Significance = * *p* 0.05, #, *p* 0.05. ROS: Reactive Oxygen Species, Nrf-2: nuclear factor erythroid 2–related factor 2,HO-1: Heme Oxygenase-1, DAPI: 4’, 6-Diamidino-2-Phenylindole, Dihydrochloride.

Oxidative stress is a key factor of AD, and several studies have indicated that Aβ deposition in AD is associated with the generation of reactive oxygen species and oxidative stress [34,35]. Nuclear factor erythroid 2-related factor 2 (Nrf2) is a cytoprotective factor with a protective role against oxidative stress. Nrf2 also regulates the expression of heme oxygenase (HO1), which removes the toxic heme from the cell and plays a protective role against oxidative stress [36,37]. To analyze the effects of lupeol on Nrf2 and HO1, we performed western blot analysis, which showed a reduced expression of Nrf2/HO1 in Aβ_1–42_-induced AD mice brains (cortex and hippocampus) compared to the saline-treated control mice. Treatment with lupeol significantly increased the expression of Nrf2/HO1 (Figure 3a). Similarly, the immunofluorescence analysis also suggested a reduced expression of Nrf2 in Aβ_1–42_-injected mice, which was significantly upregulated with the administration of lupeol, as shown in Figure 3b. To further strengthen our findings, we performed the ROS assay, which showed that the injection of Aβ_1–42_ significantly increased the level of ROS compared to the saline-treated control group. Additionally, this effect was significantly reduced with the administration of lupeol to the treated group (Figure 3c).

### 3.3. Lupeol Treatment Attenuated Aβ-Induced Glial Cells in the Brains of Mice

**Figure 4:**
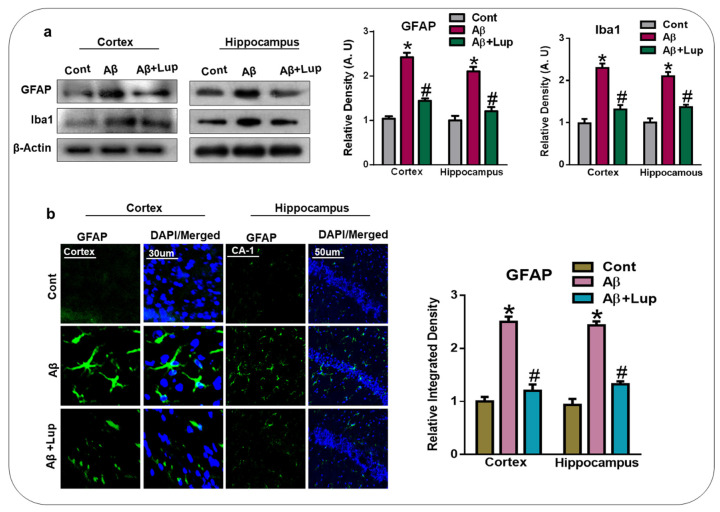
Lupeol treatment attenuated Aβ-induced glial cell in mice brain. (**a**) Western blot analysis shows the increased expression of Iba1 and GFAP in the Aβ-injected mice whereas lupeol treatment, reduced the expression of these markers (n = 6 mice/group). The bands were quantified using ImageJ software, and the differences are represented by a histogram. β-actin was used as a loading control. The density values are expressed in arbitrary units (A.U) as the means ± SEM for the respective indicated cortex and hippocampus proteins (n = 6 mice/group). (**b**) Immunofluorescence analysis GFAP (green) along with their respective histogram stained with DAPI (blue) in cortex and hippocampus (CA1 region) in the adult mice (n = 6 mice/group). The data are presented relative to control. Magnification 10×. Scale bar = 50 µm. The expressed data are relative to the control. * significantly different from saline-injected; # significantly different from Aβ-injected. Significance = * *p* 0.05, #, *p* 0.05.

Aβ deposition and oxidative stress in the brain are responsible for the activation of astrocytes and microglial cells [38]. Activated astrocytes and microglia are the main players in neuroinflammation and neurodegeneration [39]. Ionized calcium-binding adaptor molecule-1 (Iba-1) and the glial fibrillary acidic protein (GFAP) are specific markers of activated microglia and astrocytes, respectively. Therefore, we analyzed the expression of Iba-1 and GFAP in the cortex and hippocampus of the experimental mice. Our results showed an elevated expression of Iba-1 and GFAP in the Aβ_1–42_-injected mice brains (cortex and hippocampus) compared to the saline-treated control mice. Interestingly, lupeol treatment significantly decreased the expression of activated Iba-1 and GFAP in the cortex and hippocampus of experimental mice (Figure 4a). We also evaluated the expression of GFAP through immunofluorescence, which showed that Aβ_1–42_ administration increased the expression of GFAP in the cortex and hippocampus compared to the saline-treated control mice. Treatment with lupeol significantly decreased the immunoreactivity of GFAP compared to the Aβ_1–42_-injected mice (Figure 4b).

### 3.4. Oral Administration of Lupeol Reduced the Release of Inflammatory Cytokines in Aβ_1–42_-Injected Mice

**Figure 5:**
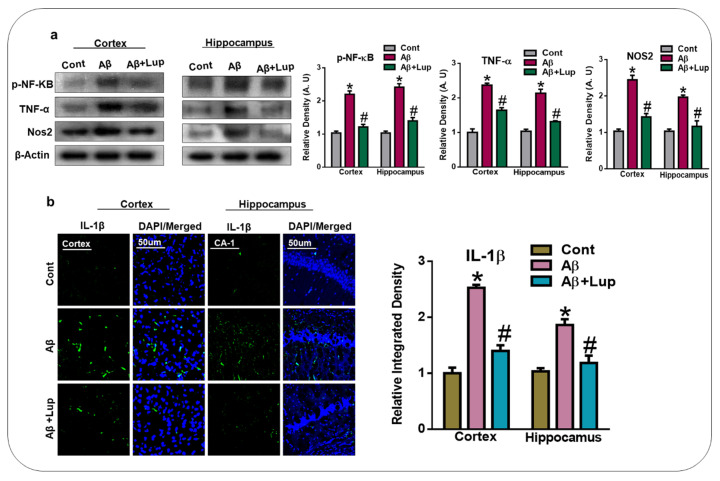
Oral administration of lupeol reduced the release of inflammatory cytokines. (**a**) Western blot analysis of inflammatory cytokines (p-NF-KB, TNF-α, and NOS2), in the cortex and hippocampus of experimental mice (n = 6 mice/group). Western blot bands were quantified by ImageJ software, and the differences were represented by a histogram. β-actin was used as a loading control. (**b**) Immunofluorescence analysis of IL-1β (green) along with their respective histogram stained with DAPI (blue) in cortex and hippocampus (CA-1 region) in the adult mice (n = 6 mice/group). The data are presented relative to the control. Magnification 10×. Scale bar = 50 µm. The expressed data are relative to the control. * Significantly different from saline-injected; # significantly different from Aβ-injected. Significance = * *p* 0.05, # *p* 0.05. NF-kB: Nuclear Factor kappa-light-chain- B, TNF-α: Tumor necrosis factor alpha, NOS-2: Nitric oxide synthase 2, IL-1β: Interleukin 1 beta.

It has been reported that activated glial cells and Aβ deposition in the brain are responsible for the release of several inflammatory markers and mediators [40,41]. Therefore, in this study, we evaluated the expressions of p-NF-κB, TNF-α, and NOS-2 in the cortex and hippocampus of adult mice of the experimental groups, which showed an upregulation of p-NF-κB, TNF-α, and NOS-2 in the Aβ_1–42_-induced mice compared to the vehicle-treated control mice. Interestingly, the expressions of these activated cytokines were significantly downregulated with the treatment of lupeol (Figure 5a). We also examined the expression of IL-1β through confocal microscopy. Our result indicated an increased immunoreactivity of IL-1β in the cortex and hippocampus of the Aβ-mouse model of AD compared to the control mice. Interestingly, the expression of IL-1β was markedly reduced with the administration of lupeol (Figure 5b). These results showed that lupeol plays an important role against inflammation by suppressing these inflammatory mediators and cytokines in the Aβ_1–42_-mouse model of AD.

### 3.5. Lupeol Treatment Enhanced Memory Impairments in Aβ_1–42_-Induced AD Mice

**Figure 6:**
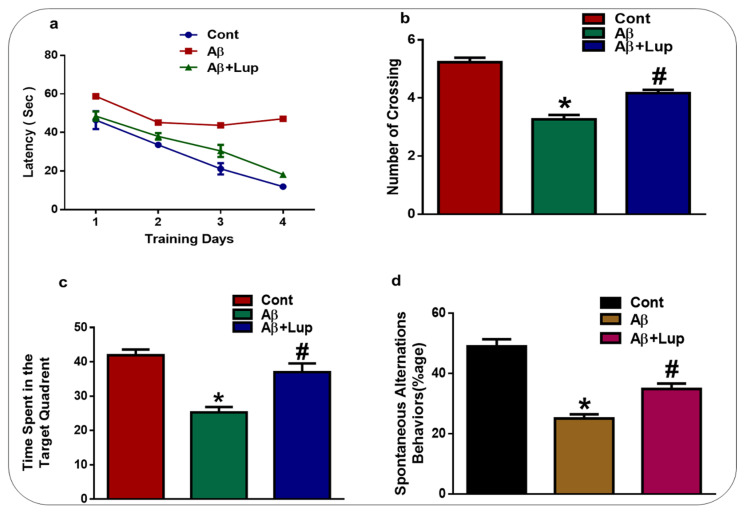
Lupeol treatment enhanced memory impairments in the Aβ-mouse model of AD. To examine the memory function of the experimental mice, the MWM and Y-maze were performed (n = 15 mice/group). (**a**) Average escape latency time for experimental mice to reach the hidden platform. (From day 1 to 4 days). (**b**) The average number of crossing in the MWM in the hidden platform during the probe test. (**c**) Time in the quadrant was previously the hidden platform was placed during the training session. (**d**) Spontaneous alteration behavior % of the mice during the Y-maze test. The data are shown as a mean ± S.E.M. * Significantly different from normal saline-treated mice and # significantly different from Aβ-injected mice, respectively; * *p* 0.05, # *p* 0.05. Cont: Control, Aβ: Amyloid beta, Lup: Lupeol.

To examine the effects of lupeol on learning and memory dysfunctions in Aβ_1–42_-injected mice, we performed the Morris water maze and Y-maze tests. In MWM, after the initial training, the animals were allowed to find the hidden platform and the latency time was recorded in the MWM task. In the training session, the Aβ_1–42_-induced mice showed memory impairments, as it took them longer to find the hidden platform compared to the saline-treated control mice (Figure 6a). After the training session, a probe test was performed, in which the hidden platform was removed, which showed that the Aβ_1–42_-injected mice spent less time in the target quadrant, while lupeol-treated mice improved in terms of the time in the target quadrant as well as the number of crossings in the area where the previously hidden platform was present (Figure 6b,c). The Y-maze result showed that Aβ_1–42_-injected mice exhibited short-term spatial memory impairments, while treatment with lupeol enhanced the percentage of spontaneous alteration behavior, which resulted in an increased function of the spatial working memory (Figure 6d). All of these results showed that lupeol treatment improved learning and memory in the Aβ-mouse model of AD.

## 4. Discussion

In the present study, we investigated the neuroprotective mechanism of lupeol in the Aβ-mouse model of AD, which suggested that lupeol suppressed the elevated oxidative stress, neuroinflammation, and memory and cognitive dysfunctions. The pathogenesis of AD occurred due to the accumulation of toxic Aβ-peptide in the central nervous system, causing synaptic dysfunction, neuronal cell death, and memory and cognitive impairments [42,43]. The amyloid precursor protein (APP) can be cleaved by the beta-amyloid cleaving enzyme (BACE-1), which accelerates the production of Aβ-peptide in the brain. BACE-1 is a potential target for the prevention and treatment of AD [44,45]. In our findings, the level of Aβ and BACE-1 in the Aβ_1–42_-mouse model of AD was significantly reduced with the administration of lupeol, as shown by the western blot and immunofluorescence analysis (Figure 2a,b). Inhibition of the amyloidogenic process may be achieved by different mechanisms, one of which is rescuing the brains against elevated oxidative stress [46]. Oxidative stress is a key factor of AD, and several studies have indicated that Aβ deposition in AD is associated with the generation of reactive oxygen species and oxidative stress [47]. Nuclear factor erythroid 2-related factor2 (Nrf2) is a member of the cap ‘n’ colon family, which is a master regulator of oxidative stress. It plays a protective role against oxidative stress, and also regulates several other signaling pathways and important anti-oxidant genes [48,49]. Heme oxygenase (HO1) is the target gene of Nrf2, which removes toxic heme, carbon oxide, and iron, and plays a protective role against oxidative injury [48]. The effects of lupeol against the elevated oxidative stress indicates that lupeol may reduce the amyloidogenic process by reducing the oxidative stress, as indicated previously [50]. To unveil this, we examined the oxidative stress-related parameters in the experimental groups, which suggested that lupeol markedly reduced the elevated oxidative stress compared to the Aβ_1–42_-injected mice (Figure 3a–c). To analyze the effects of lupeol against the amyloid-beta-induced activated astrocytes and microglia, we checked the expression of Iba-1 and GFAP in the experimental groups, which showed the reduced expression of these markers with the administration of lupeol (Figure 4a,b). The activated microglial cells further aggravated the phosphorylation and nuclear translocation of p-NF-κB, which further facilitated the release of inflammatory cytokines and mediators [51]. The p-NF-κB is a large family of innate immunity and a major regulator in the initiation of inflammation [52]. Tumor necrosis factor-alpha (TNF-α) is a potent pro-inflammatory cytokine that ameliorates neuroinflammation in neurodegenerative diseases [53]. Nitric oxide 2 (NOS-2) plays an important role in neuroinflammation by generating nitric oxide (NO), and the excessive NO production is one of the major causative reagents of neuroinflammation and neurodegeneration [54]. Therefore, we checked, through western bolt analysis, the expression of p-NF-κB, TNF-α, and NOS-2 in the experimental groups. The result showed the elevated expression of these inflammatory cytokines in the cortex and hippocampus of Aβ_1–42_-injected mice, while treatment with lupeol decreased the expression of these inflammatory mediators (Figure 5a). Furthermore, confocal microscopy showed the increased immunoreactivity of IL-1β in the Aβ-mouse model of AD; however, treatment with lupeol reduced the expression of IL-1β in the experimental animals (Figure 5b). The overall findings suggested that lupeol suppressed the activated microglial cells and inflammatory mediators, and thereby conferred neuroprotection to the brains of mice against Aβ_1–42_-induced neuroinflammation. Aβ accumulation, oxidative stress, and inflammation in the brain accelerate the cognitive and spatial working memory [55]. Therefore, we performed the MWM and Y-maze tests, which suggested that with the inhibition of oxidative stress and neuroinflammation, there was a significant improvement in the cognitive functions of the mice (Figure 6a–c). Similarly, in the Y-maze test, the Aβ_1–42_-injected mice exhibited a lower percentage of spontaneous alternation behaviors, while lupeol enhanced the spontaneous alteration behavior and reduced the spatial working memory (Figure 6d). The overall findings are in accordance with our previous study conducted on lupeol, where we demonstrated that lupeol suppresses neuroinflammation [28].

## 5. Conclusions

In conclusion, we suggest that lupeol has strong anti-oxidant, anti-neuroinflammatory, and anti-amyloidogenic effects. Moreover, this study also indicated that lupeol reverses the memory deficits in the Aβ-mouse model of AD. Based on current and previous studies, lupeol may protect the brains of mice against Aβ-induced oxidative stress-mediated neuroinflammation and cognitive dysfunctions. Our findings may be fruitful for the advancement of new therapeutic approaches for the management of AD-like conditions.

## Figures and Tables

**Table 1:** Information on the primary antibodies.

Name	Source	Application	Manufacturer	Catalog Number	Concentration
**Aβ**	Mouse	WB/IF	Santa Cruz Biotechnology, United States	SC: 28365	1:1000/1:100
**Bace-1**	Mouse	WB	=	SC: 33711	1:1000
**Nrf-2**	Mouse	WB/IF	=	SC: 365949	1:1000/1:100
**HO-1**	Mouse	WB	=	SC: 136961	1:1000
**GFAP**	Mouse	WB/IF	=	SC: 33673	1:1000/1:100
**Iba-1**	Rabbit	WB	abcam	Ab: 178846	1:1000
**P-NF-kB**	Mouse	WB	Santa Cruz Biotechnology, United States	SC: 136548	1:1000
**TNF-α**	Mouse	WB	=	SC: 52746	1:1000
**NOS-2**	Rabbit	WB	=	SC: 651	1:1000
**IL-1β**	Mouse	IF	=	SC: 32294	1:100
